# The C-Terminal Domain of the Arabinosyltransferase *Mycobacterium tuberculosis* EmbC Is a Lectin-Like Carbohydrate Binding Module

**DOI:** 10.1371/journal.ppat.1001299

**Published:** 2011-02-24

**Authors:** Luke J. Alderwick, Georgina S. Lloyd, Hemza Ghadbane, John W. May, Apoorva Bhatt, Lothar Eggeling, Klaus Fütterer, Gurdyal S. Besra

**Affiliations:** 1 School of Biosciences, University of Birmingham, Edgbaston, Birmingham, United Kingdom; 2 Institut für Biotechnologie I, Forschungszentrum Jülich, Jülich, Germany; Johns Hopkins School of Medicine, United States of America

## Abstract

The d-arabinan-containing polymers arabinogalactan (AG) and lipoarabinomannan (LAM) are essential components of the unique cell envelope of the pathogen *Mycobacterium tuberculosis*. Biosynthesis of AG and LAM involves a series of membrane-embedded arabinofuranosyl (Ara*f*) transferases whose structures are largely uncharacterised, despite the fact that several of them are pharmacological targets of ethambutol, a frontline drug in tuberculosis therapy. Herein, we present the crystal structure of the C-terminal hydrophilic domain of the ethambutol-sensitive Ara*f* transferase *M. tuberculosis* EmbC, which is essential for LAM synthesis. The structure of the C-terminal domain of EmbC (EmbC^CT^) encompasses two sub-domains of different folds, of which subdomain II shows distinct similarity to lectin-like carbohydrate-binding modules (CBM). Co-crystallisation with a cell wall-derived di-arabinoside acceptor analogue and structural comparison with ligand-bound CBMs suggest that EmbC^CT^ contains two separate carbohydrate binding sites, associated with subdomains I and II, respectively. Single-residue substitution of conserved tryptophan residues (Trp868, Trp985) at these respective sites inhibited EmbC-catalysed extension of LAM. The same substitutions differentially abrogated binding of di- and penta-arabinofuranoside acceptor analogues to EmbC^CT^, linking the loss of activity to compromised acceptor substrate binding, indicating the presence of two separate carbohydrate binding sites, and demonstrating that subdomain II indeed functions as a carbohydrate-binding module. This work provides the first step towards unravelling the structure and function of a GT-C-type glycosyltransferase that is essential in *M. tuberculosis*.

## Author Summary

Tuberculosis (TB), an infectious disease caused by the bacillus *Mycobacterium tuberculosis*, burdens large swaths of the world population. Treatment of active TB typically requires administration of an antibiotic cocktail over several months that includes the drug ethambutol. This front line compound inhibits a set of arabinosyltransferase enzymes, called EmbA, EmbB and EmbC, which are critical for the synthesis of arabinan, a vital polysaccharide in the pathogen's unique cell envelope. How precisely ethambutol inhibits arabinosyltransferase activity is not clear, in part because structural information of its pharmacological targets has been elusive. Here, we report the high-resolution structure of the C-terminal domain of the ethambutol-target EmbC, a 390-amino acid fragment responsible for acceptor substrate recognition. Combining the X-ray crystallographic analysis with structural comparisons, site-directed mutagenesis, activity and ligand binding assays, we identified two regions in the C-terminal domain of EmbC that are capable of binding acceptor substrate mimics and are critical for activity of the full-length enzyme. Our results begin to define structure-function relationships in a family of structurally uncharacterised membrane-embedded glycosyltransferases, which are an important target for tuberculosis therapy.

## Introduction

**Figure 1:**
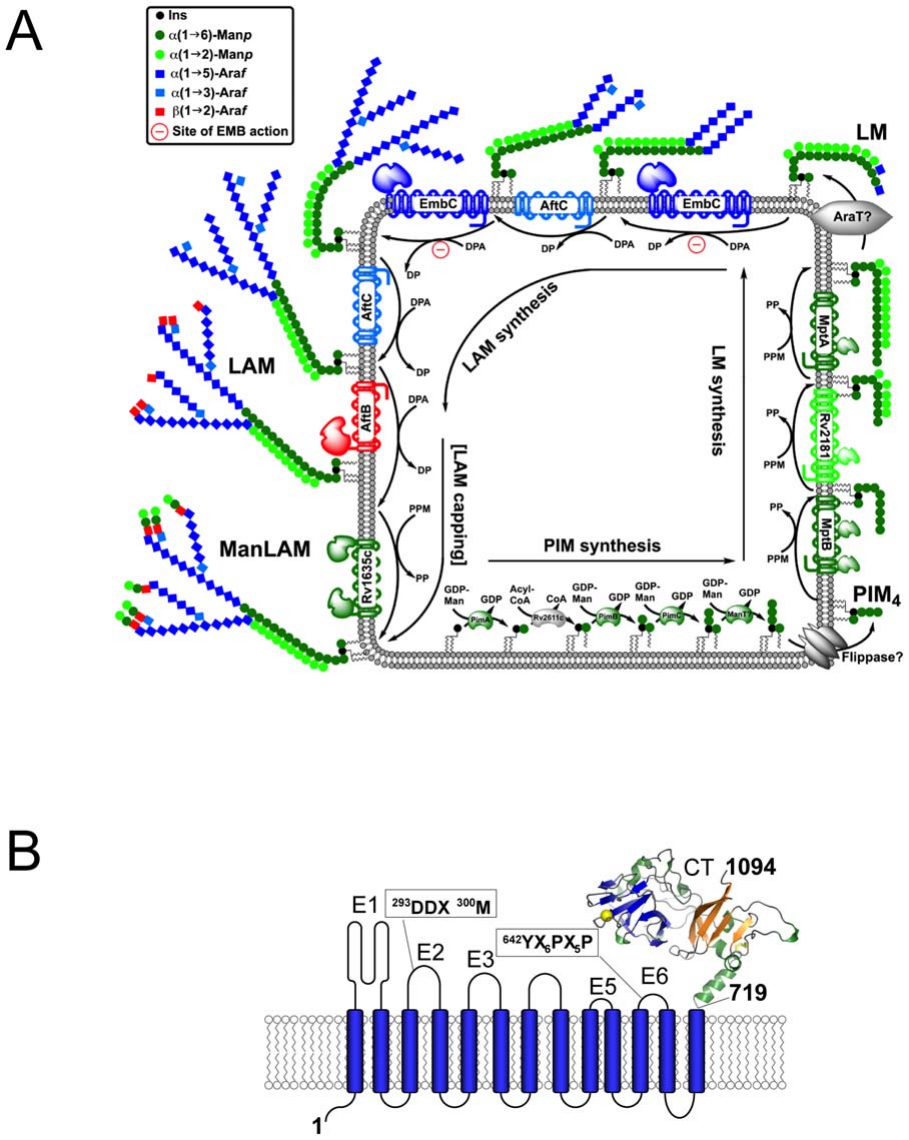
Schematic diagram of LAM synthesis and architecture of *M. tuberculosis* EmbC. A) Schematic representation of the stepwise assembly of LAM at the membrane of mycobacteria. The precursors of LAM are phosphatidylinositol mannosides (PIM), which contain a phosphatidyl-*myo*-inositol core unit. Initially, intracellular α-mannosyltransferases catalyse attachment of mannosyl units to inositol, followed by flipping of the glycolipid to the extracellular face of the membrane and further chain extension by membrane-embedded mannosyl- and arabinofuranosyl transferases to generate lipomannan (LM), lipoarabinomannan (LAM) and mannan-capped LAM (ManLAM). Relevant saccharide donor substrates are as follows: GDP-Man (guanosine-5′-diphosphate-α-D-mannose), PPM (C35/C50-polyprenyl-monophospho-mannose), DPA (β-D-arabinofuranosyl-1-monophosphoryl-decaprenol). ManT and AraT designate mannosyl- and arbinosyltransferases that are as yet uncharacterised. B) Topology diagram of EmbC based on the hydropathy analysis with TMHMM (www.cbs.dtu.dk/services/TMHMM/). Extracellular loops are labelled E1-E6 and CT, intracellular loops I1–I7. Functionally important sequence motifs, previously identified in references [10], [15], are indicated. The C-terminal domain (residues 719–1094) is shown as a ribbon diagram.

Tuberculosis (TB) affects large parts of the world's population, particularly in developing countries [1]. The antibiotics isoniazid (INH) and ethambutol (EMB) [2] have been used for decades as frontline drugs to treat *Mycobacterium tuberculosis* infections, the causative agent of TB, but the rise of multi-drug resistant (MDR) and extensively drug resistant (XDR) strains poses a serious threat to present treatment options [3]. Both, INH and EMB inhibit the synthesis of essential components of the mycobacterial cell wall. This unique and highly impermeable barrier surrounds a single phospholipid bilayer membrane and is composed of an outer segment of solvent-extractable lipids, glycans and proteins, and a covalently linked inner segment, known as the mycolyl-arabinogalactan-peptidoglycan (mAGP) core [4]. Perturbations to the mAGP core tend to undermine viability of *M. tuberculosis*, a major reason why mAGP biosynthesis constitutes an attractive target for drug design efforts. The mycobacterial cell wall also encompasses various membrane-anchored lipoglycans, a group that includes lipoarabinomannan (LAM), which plays a key role in modulating the host immune response [5]. The arabinogalactan (AG) segment of the mAGP core and LAM both contain d-arabinan polymer, composed of α(1→5), α(1→3) and β(1→2)-linked arabinofuranosyl (Ara*f*) residues that are assembled in distinct structural motifs (Fig. 1A) [4], [5].

In recent years, substantial progress has been made in defining the enzymatic processes resulting in the complete synthesis of AG and LAM [6]–[14]. Probing susceptibility to EMB, initial studies established that this inhibitor acted on a set of closely related arabinofuranosyl (Ara*f*) transferases, EmbC (Rv3793), EmbA (Rv3794) and EmbB (Rv3795) [6], [7], collectively referred to as the Emb enzymes. These three proteins belong to the glycosyltransferase superfamily C (GT-C), which encompasses a diverse set of membrane-embedded glycosyltransferases that utilise lipid-linked as opposed to nucleotide-linked sugars as donor substrates (Fig. 1A) [15]. The Emb enzymes of *M. tuberculosis* display a common architecture of 13 transmembrane helices in conjunction with a hydrophilic C-terminal domain [10], [14] (Fig. 1B), and share the same polyprenyl donor-substrate, β-D-arabinofuranosyl-1-monophosphoryldecaprenol (DPA) [16], [17].

Owing to their hydrophobic nature, generating recombinant Emb proteins in soluble form has proved difficult, hampering *in vitro* characterisation. As a result, the function of the Emb enzymes has been delineated by genetics, phenotypic analysis of the cell envelope and cell-free assays. Single gene deletions of *embC*, *embB* in *M. tuberculosis* are lethal [18], [19], but corresponding knock-outs in *Mycobacterium smegmatis* or *Corynebacterium glutamicum* yield viable, albeit slow growing mutants, whose cell wall defects can be analysed [8], [9]. Following attachment of the initial Ara*f* residue to the linear galactan polymer [Gal*f*-β(1→5)Gal*f*-β(1→6)]_n_, catalysed by the Ara*f*-transferase AftA [12], EmbA and EmbB extend the arabinan chain in AG synthesis, transferring Ara*f* residues from DPA to polysaccharide acceptors [8], [9]. Highly similar in amino acid sequence (∼40% identity, see also Supporting Fig. S1), EmbA,B and EmbC have differential roles: the *ΔembA,B* deletions inhibit AG synthesis, but leave LAM synthesis intact, whereas the *ΔembC* deletion only affects LAM synthesis. Chimaeric forms of the Emb enzymes, where the hydrophilic C-terminal domain of EmbC was swapped for that of EmbB led to a hybrid-LAM, bearing an AG-specific, branched Ara*f*_6_ group instead of the characteristic LAM-specific linear Ara*f*_4_ [9]. These data indicated that the hydrophilic C-terminal domain makes a critical contribution to determining the structure of the resulting AG or LAM segments.

To date, the Emb enzymes have remained poorly characterised in structural terms, despite their central significance as targets of the TB antibiotic EMB and their link to drug resistance [20]. Herein, we present the crystal structure of the C-terminal hydrophilic domain of *M. tuberculosis* EmbC (residues 719–1094, henceforth EmbC^CT^), as a first step towards the elucidation of the 3D structure of the full-length enzyme.

## Results

### Structure determination and domain architecture

**Figure 2:**
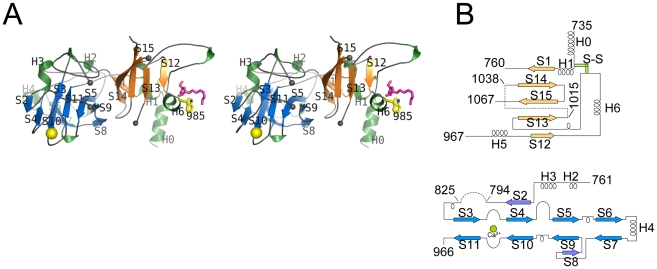
Stereo diagram of EmbC^CT^ and topology of its subdomains. A) Stereo ribbon diagram of EmbC^CT^ with definition of the secondary structure elements. Grey spheres indicate the boundaries of the disordered loops. The Ca^2+^ ion (yellow sphere), and positions of Trp985 (yellow sticks) and of the Ara(1→5)Ara-O-C8 ligand (magenta) are shown. B) Topology diagrams of subdomains I (top) and II (bottom), illustrating the connectivity of secondary structure elements and the jelly roll topology of subdomain II.

**Figure 3:**
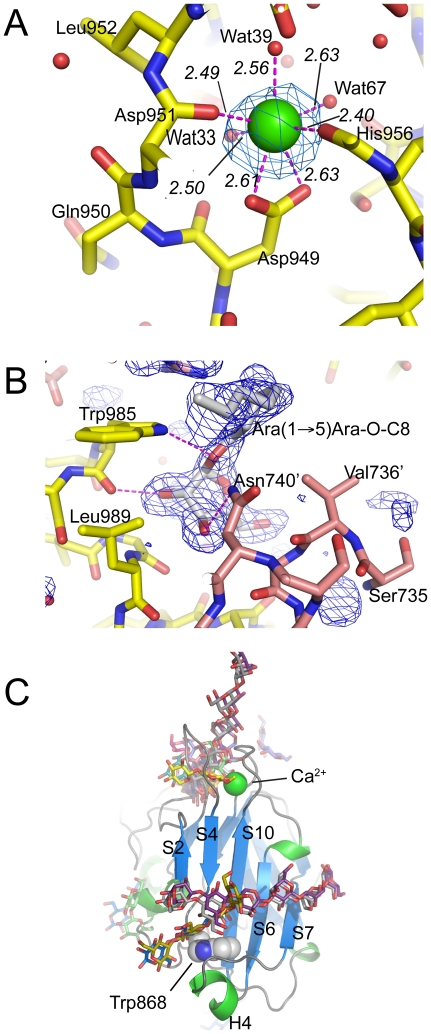
Metal binding and putative carbohydrate binding sites. A) Ca^2+^ site (green sphere) superimposed with an anomalous difference density map (3σ contour level) calculated with in-house diffraction data (CuKα radiation). Metal-ligand interactions are indicated with distances in units of Å. B) σ_A_-weighted *F_o_−F_c_* difference density map (3σ contour level) of the Ara(1→5)Ara-O-C8 binding site calculated with phases and calculated amplitudes *F_c_* of the model coordinates prior to incorporation of the ligand. Two symmetry-related molecules are shown (yellow and pink sticks, respectively). Primed residue numbers refer to the symmetry mate. C) Identification of putative carbohydrate binding sites in subdomain II by superimposing EmbC^CT^ with carbohydrate–bound structural homologues. Ligands (shown as stick models) were drawn according to the DALI-alignment with the Cα-traces of structural neighbours. Ligand structures shown in this diagram encompass PDB entries 1ux7, 1w9t, 1o8s, 1w9w, 1uy2, 1od3, 2vzq, 2w47, 2w87, 2cdp, 2cdo, 1uyy, 1uy0, representing the top 10 matches of the DALI search against the PDB90 subset (chains that are less than 90% identical in sequence to each other; *Z*-scores 6.9–6.3, *RMSD* 3.0–3.6 Å).

EmbC^CT^ crystallised in space group *P*6_5_22 over a diverse range of reservoir conditions, with one molecule in the crystallographic asymmetric unit. Crystals were generated with or without an Ara*f* acceptor analogue (see below) present in the crystallisation droplet. The experimental density, phased by multi-wavelength anomalous dispersion (2.7 Å, Table 1), was of very good quality (Fig. S2A), defining the structure for residues 735–1067, except for two disordered loops (795–824 and 1016–1037, Fig. 2A). EmbC^CT^ is composed of two distinct subdomains, separated by a deep crevice marked by the disordered loops (residues 795–824 and 1016–1037). Subdomain I, which encompasses residues 746–760 and 967–1067, displays a mixed α/β structure, with a 5-stranded β-sheet forming a semi-barrel (Fig. 2A). The long H6-S13 loop, which forms a minor crystal packing interface, protrudes from the core of subdomain I with a helical half-turn at its tip (Fig. 2A). Subdomain II (residues 761–966) forms an anti-parallel β-sandwich structure, of which the ‘outer’ sheet (S2, S4, S10, S6, S7) faces solvent while the ‘inner’ sheet (S3, S11, S5, S9, S8) packs against the core of the domain (Fig. 2A). The β-sandwich of subdomain II assumes a jellyroll fold (Fig. 2B), a fold typical for polysaccharide binding units in plant lectins and carbohydrate active enzymes [21]. Although not part of the formal jellyroll description, strands S2 and S8 extend the ‘outer’ and ‘inner’ sheet, respectively, while helix H4 forms a boundary to the ‘outer’ sheet. A high-density peak (14σ, anomalous density difference map, Fig. 3A) is embedded between loops S3–S4 and S10–S11. Quasi-octahedral coordination geometry and the distribution of peak-ligand distances from 2.40 to 2.63 Å (Fig. 3A) suggest a bound Ca^2+^ ion [22]. The metal ion appears shielded from solvent, although including 10 mM EDTA in the cryoprotectant buffer significantly diminished the height of the density peak (Fig. S2B). Substitution of Asp949 by serine in EmbC^CT^, the only side chain in direct contact with the Ca^2+^ ion (2.6 Å, bidentate, Fig. 3A), resulted in very poor recombinant expression compared to wild-type and other point mutants probed in this study (see below). Together these observations suggest that the Ca^2+^ ion is important for the structural integrity of EmbC^CT^.

**Table 1:** Crystallographic data.

Data collection				
Data set	Native	SeMet-IP	SeMet-Peak	SeMet-Remote
Wavelength (Å)	0.9763	0.9799	0.9797	0.9763
Space group	*P*6_5_22	*P*6_5_22	*P*6_5_22	*P*6_5_22
Unit cell: a, c (Å)	129.1, 136.7	130.3, 137.7	130.3, 137.7	130.3, 137.7
Resolution(1)	46.9−2.0 (2.11−2.0)	29.5−2.7 (2.85−2.7)	29.5−2.7 (2.85−2.7)	29.5−2.7 (2.85−2.7)
Completeness(1)	100 (100)	99.9 (100)	99.9 (100)	99.5 (97.9)
Rsym (%)(1)	7.3 (56.4)	11.1 (41.8)	11.2 (42.4)	5.8 (12.9)
I/σ(I)(1)	27 (5.3)	20.9 (7.1)	20.4 (6.9)	32.4 (19.1)
Redundancy(1)	14.4 (14.5)	17.6 (18.0)	17.6 (18.1)	17.5 (17.7)

Refinement	
Resolution	43.3−2.0
Unique Reflections	86,163
Protein residues	285
Ara(1→5)Ara-O-C8	1
Ca^2+^	1
PO_4_^3−^	1
Water molecules	113
Rwork, Rfree(2) (%)	18.7, 20.7
Wilson B-factor (Å^2^)	27.9
Aver. B-factor (all, Å^2^)	32.3
Protein (Å^2^)	32.2
Ara(1→5)Ara-O-C8 (Å^2^)	46.1
Solvent (Å^2^)	34.7
RMSD B-factors (Å^2^)	2.3, 2.5
RMSD bonds (Å)	0.006
RMSD angles (°)	1.02
Ramachandran plot(3)	
Favoured (%)	97.8
Outliers	1 of 278

(1) High resolution shells are given in parentheses.

(2) 5% of reflections were set aside for the test set.

(3) Ramachandran statistics was calculated using MolProbity (molprobity.biochem.duke.edu; [47]).

### Structural neighbours

The fold of subdomain II is consistent with the proposed role of EmbC^CT^ as an acceptor saccharide recognition module. The comparison with structural homologues, identified via distance matrix alignment using the DALI program (http://ekhidna.biocenter.helsinki.fi/dali_server/, [23]) reinforces this notion. The vast majority of PDB entries retrieved by DALI (over 300 entries above the default significance threshold of *Z* = 2) match the β-sandwich fold of subdomain II and represent ‘carbohydrate binding modules’ (CBM), structural domains that confer carbohydrate-binding specificity, but that lack intrinsic catalytic activity [21]. CBMs occur frequently as a part of glycoside hydrolase enzymes and fall into (to date) 61 distinct CBM families (http://www.cazy.org/). While none of the structural homologues is particularly close to subdomain II (*Z*-scores≤6.9, root mean square deviation (*RMSD*)≥3.0 Å), the top 10 hits include the calcium-containing CBM families 6 and 36 (Fig. S3A–C). Interestingly, in the DALI-generated superposition of EmbC^CT^ with *Paenibacillus polymyxa* endo-1,4-β-xylanase (PDB entry 1UX7, CBM 36), the Ca^2+^ sites match to within 0.9 Å, and in the latter, the Ca^2+^ ion makes direct contact with the bound xylobiose ligand (Fig. S3A). In contrast, only three hits were obtained for subdomain I of which only the best (PDB entry 2ZAG, *Z* = 3.0, RMSD 3.4 Å for 66 Cα pairs) showed weak similarity in terms of secondary structure topology in a limited region of overlap (Fig. S4). This PDB entry describes the hydrophilic C-terminal domain of oligosaccharyltransferase STT3 from *Pyrococcus furiosus* [24], a membrane-embedded glycosyltransferase of the GT-C superfamily that catalyses transfer of glycosyl groups from a lipid donor to Asn-glycosylation sites of the acceptor protein.

### Self-assembly in solution

**Figure 4:**
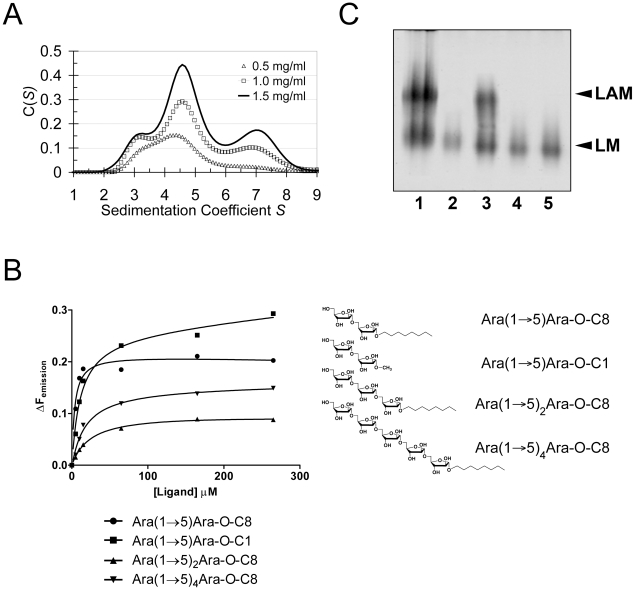
Self-assembly, ligand binding and cell wall analysis. A) Self-assembly of EmbC^CT^ by analytical ultracentrifugation in sedimentation velocity mode. Protein concentration for the individual distributions is given in units of mg ml^−1^. Peaks at 3.1*S*, 4.6*S* and 7*S* correspond to fitted molecular weights of 46500 Da, 75800 Da and 138000 Da, respectively. B) Saturation binding of arabinofuranosyl acceptor analogues to EmbC^CT^ probed by intrinsic tryptophan fluorescence. The chemical structures of the ligands are indicated. Data points were fitted to a single site-binding model. C) Effect of substitutions W868A and W985A in full-length *M. tuberculosis* EmbC on *in vivo* lipomannan (LM) and LAM synthesis analysed by SDS-PAGE. Lanes are as follows: (1) *M. smegmatis* wild-type; (2) *M. smegmatis* Δ*embC*; (3) *M. smegmatis* Δ*embC*+pVV16-Mt-embC; (4) *M. smegmatis* Δ*embC*+pVV16-Mt-embC^W868A^; (5) *M. smegmatis* Δ*embC*+pVV16-Mt-embC^W985A^.

Crystal packing contacts, analysed using the PISA server (http://www.ebi.ac.uk/msd-srv/prot_int/pistart.html), highlighted three prominent interaction surfaces burying 390 Å^2^, 670 Å^2^ and 1100 Å^2^ of solvent accessible surface (SAS) per monomer, respectively (Fig. S5). We probed self-assembly of EmbC^CT^ by sedimentation velocity at three different protein concentrations (Fig. 4A). The distribution *C(S)* of the sedimentation coefficient *S* indicates a dynamic equilibrium between three different molecular species at 3.1*S*, 4.6*S* and 7*S*, which correspond to apparent molecular weights of 46.5 kDa, 75.8 kDa and 138.0 kDa, respectively, compared to the calculated monomer mass of 39.9 kDa. Bearing in mind that under- or overestimates of apparent masses can occur as a result of fitting a single frictional coefficient for an ensemble of species with different frictional ratios, the dominant peak at 4.6*S* most likely represents a dimer. The higher molecular weight peak at 7.6*S*, could be a trimer or tetramer, but strongly suggests that more than one of the crystal packing interfaces is able to mediate oligomerisation of EmbC^CT^ *in vitro*.

### Carbohydrate binding

Previous studies had attributed to the C-terminal domain of the Emb proteins a critical role in arabinan chain extension [9], [11]. Therefore, we asked whether the isolated domain is able to bind synthetic acceptor analogues. As the physiological substrate is chemically complex and diverse, using synthetic acceptor analogues offered the best chance to obtain an experimental acceptor-bound complex structure. In previous work, our laboratory had chemically synthesised neo-glycolipid acceptors that were modelled on motifs found in mycobacterial AG and LAM. When incubated with [^14^C]-labelled Ara*f*-donor substrate DPA and isolated mycobacterial membranes in a cell-free Ara*f* transferase, these molecules acted as potent acceptor mimics [25]. One of these acceptors was the di-arabinoside α-D-Ara*f*-(1→5)-α-D-Ara*f*-*O*-(CH_2_)_7_CH_3_ (for short: Ara(1→5)Ara-O-C8, Fig. 4B). The *O*-linked octyl tail allowed extraction of the reaction products for qualitative characterisation *in vitro*. Importantly, the closely related di-arabinoside α-D-Ara*f*-(1→5)-α-D-Ara*f*-*O*-CH_3_ (Ara(1→5)Ara-O-C1) exhibited similar levels of acceptor activity, demonstrating the *O*-linked octyl was dispensable for activity [25]. By way of intrinsic tryptophan fluorescence, we probed binding of Ara(1→5)Ara-O-C8 to EmbC^CT^, as well as that of analogous tri- and penta-arabinofuranosides, [α-D-Ara*f*-(1→5)]_2_-α-D-Ara*f*-*O*-(CH_2_)_7_CH_3_ (Ara-α(1→5)_2_-Ara-O-C8) and [α-D-Ara*f*-(1→5)]_4_-α-D-Ara*f*-*O*-(CH_2_)_7_CH_3_ (Ara-α(1→5)_4_-Ara-O-C8, Fig. 4B). Fitting the binding curves to a single-site saturation model, yielded an equilibrium dissociation constant K_d_ of 3.6 µM for the di-arabinofuranoside Ara(1→5)Ara-O-C8 (Table 2), while the disaccharide lacking the octyl chain, Ara(1→5)Ara-O-C1, resulted in a K_d_ of 11.0 µM. These data confirmed that in the solution state the octyl chain is not essential for binding, although it may enhance affinity. Soaking EmbC^CT^ crystals in cryoprotectant solution containing 27 mM Ara(1→5)Ara-O-C8 (∼3-fold excess of ligand relative to protein concentration in the crystal) reproducibly resulted in defined ligand density (Fig. 3B), allowing us to unequivocally build one Ara*f* unit and the octyl chain of Ara(1→5)Ara-O-C8, while the second Ara*f* ring remained invisible, even when contouring the map at near-noise level. Soaking experiments using the other acceptor analogues, for which solution binding was examined, failed to reveal electron density for the ligand. The soaked di-arabinofuranoside ligand is positioned between two symmetry-related copies of EmbC^CT^, forming non-covalent contacts only with residues in subdomain I, but not with the CBM-like subdomain II, in contrast to our expectation. The Ara*f* moiety packs against helix H6 and the H6-S13 loop (Fig. 2), forming three direct H-bond contacts with protein: O2 binds to carbonyl O of Trp985 (2.53 Å), O1 to Nε1 of Trp985 (2.99 Å), and O3 to Nδ2 of Asn740′ (primed residues indicating the symmetry mate). In contrast, the octyl chain binds between helix H0 and the S13–S14 loop of the symmetry mate (Fig. 3B). Ligand binding promotes ordering of the N-terminus of helix H0, where 3 additional residues become visible compared to apo, and induces a conformational shift of aspartate residues 1051 and 1052 in the S13–S14 loop (Fig. S6). While this crystallographic complex structure did not reveal binding to the CBM-like subdomain II, it is possible that crystal lattice formation of EmbC^CT^ interferes with binding at a site on subdomain II. We, therefore, asked whether the structural superimposition with saccharide-bound CBM domains could be exploited to predict potential additional binding sites. We note that ligand binding modes and substrate specificity of CBM domains can differ even within the same CBM family [21], [26]. Thus, structural alignments of the protein scaffolds are unlikely to accurately predict the precise modes of binding and potential specificity-determining interactions. Nevertheless, superimposing carbohydrate-bound structures of CBM domains with the 10-highest DALI *Z*-scores (with respect to the non-redundant PDB90 subset) shows two clusters of putative ligand binding sites in subdomain II (Fig. 3C): (1) near the Ca^2+^ site and the S3–S4 loop, and (2) on the open surface of the ‘outer’ β-sheet (strands S2, S4, S10, S6, S7). Virtually all ligands in the first cluster sterically clash with the loops that coordinate the Ca^2+^ site. Without invoking a conformational change that exposes the Ca^2+^ to solvent, this site appears unable to accommodate a ligand. In contrast, in the second cluster, only minor steric hindrance occurs between EmbC^CT^ and the superimposed ligands, and thus this site appeared more plausible as a carbohydrate-binding site.

**Table 2:** Ligand binding parameters.

Protein	Ligand	*F_max_*	*K_d_* (µM)	Standard Error*
EmbC^CT^	Ara(1→5)Ara-O-C8	0.22	3.65	±1.72
	Ara(1→5)Ara-O-C1	0.26	11.03	±4.01
	Ara(1→5)_2_Ara-O-C8	0.105	25.30	±4.98
	Ara(1→5)_4_Ara-O-C8	0.157	18.02	±6.97
	Galβ(1→5)Gal-O-C8	a		
EmbC^CT(N740A)^	Ara(1→5)Ara-O-C8	0.141	26.46	±6.91
EmbC^CT(W868A)^	Ara(1→5)Ara-O-C8	0.162	9.91	±1.87
	Ara(1→5)_4_Ara-O-C8	a		
EmbC^CT(H911A)^	Ara(1→5)Ara-O-C8	0.170	3.99	±0.43
EmbC^CT(W985A)^	Ara(1→5)Ara-O-C8	a		
	Ara(1→5)_4_Ara-O-C8	0.164	12.17	±6.25

*Derived from non-linear fitting of data recorded to Δ*F_emission_* = *F_max_*×[*L*]/(*K_d_*+[*L*]) (single site saturation binding curve).

a, no change in *F_emission_* over background in response to ligand additions.

### Mutagenesis and activity in full-length EmbC

The crystallographic complex of EmbC^CT^ bound to Ara(1→5)Ara-O-C8 and the structural superposition with carbohydrate-bound homologues had indicated two distinct regions in EmbC^CT^ as potential sites for carbohydrate binding (Fig. S7A). In order to probe the relevance of these two sites, we asked whether replacement of endogenous EmbC with recombinant EmbC carrying appropriate point mutations would alter the cell wall composition of *M. smegmatis*. Aromatic residues frequently mediate binding of carbohydrate ligands to CBMs [21]. Given the H-bond contacts between Trp985 and Ara(1→5)Ara-O-C8 in subdomain I, and the central position of Trp868 of the ‘outer’ (solvent-exposed) β-sheet of subdomain II (Fig. 3C and Fig. S7A), we probed these two residues in the first instance.

Using a phage-mediated transduction method for allelic exchange [27], we generated an EmbC-deficient strain of *M. smegmatis* (*M. smegmatis ΔembC*), which was complemented with plasmids encoding either wild-type (full length) *M. tuberculosis* EmbC or mutant forms thereof. In accordance with previously reported data [9], our *M. smegmatis ΔembC* strain retains lipomannan (LM) synthesis, but is deficient in LAM (Fig. 4C – lane 2). The abrogation of LAM biosynthesis can be directly attributed to the loss of EmbC, which is involved in the early synthesis of α(1→5)-Ara*f* arabinan elongation of LM, the immediate LAM precursor (Fig. 1A) [9]. We utilised this phenotype by analysing LM/LAM resulting from complementation of *M. smegmatis ΔembC* with plasmid pVV16-Mt-*embC*, encoding full-length *M. tuberculosis* EmbC, and plasmids pVV16-Mt-*embC* ^W868A^ or pVV16-Mt-*embC* ^W985A^, which encode point mutants W868A and W985A of full-length *M. tuberculosis* EmbC, respectively. Complementation with wild type EmbC largely restored the normal phenotype (Fig. 4C – lane 3), whereas complementation with the point mutants failed to re-establish LAM synthesis (Fig. 4C – lanes 4, 5). We verified by Western blot that loss of LAM synthesis was not due to failure of the plasmid-encoded protein to incorporate into the membrane of *M. smegmatis ΔembC* (Supporting Fig. S7B). These results suggest that the structural perturbations caused by the individual single-site mutations are sufficient to disrupt the function of EmbC.

### Differential acceptor binding of EmbC^CT^ mutants

**Figure 5:**
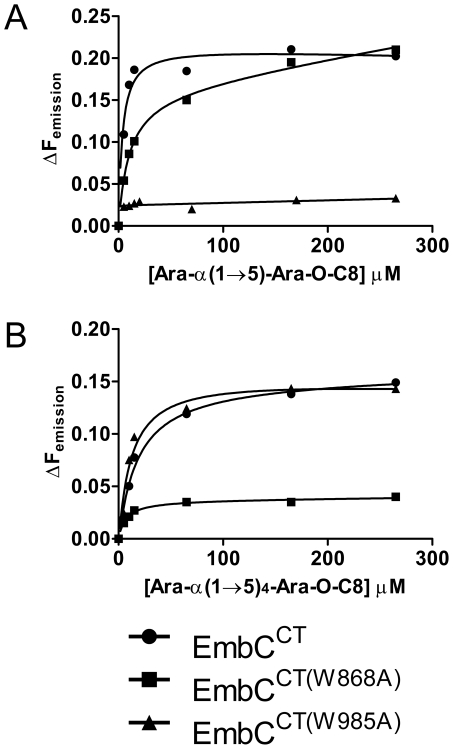
Differential binding of di- and penta-arabinofuranoside acceptor analogues to point mutants of EmbC^CT^. Ligand binding was analysed by intrinsic tryptophan fluorescence, comparing saturation binding of wild type EmbC^CT^, EmbC^CT(W868A)^ and EmbC^CT(W985A)^ for the ligands Ara(1→5)Ara-O-C8 (panel A) and Ara(1→5)_4_Ara-O-C8 (panel B). Equilibrium dissociation constants derived from non-linear fitting are reported in Table 2.

In order to establish whether loss of activity was linked to compromised acceptor binding, we introduced the single-residue mutations W868A or W985A into expression plasmids encoding EmbC^CT^. In addition, we prepared analogous expression plasmid constructs bearing mutations on Asn740 (to Ala, binding site subdomain I), Gln899 (to Ser) and His911 (to Ala, binding site subdomain II) and Asp949 (to Ser, Ca^2+^ binding site, see Supporting Fig. S7A). Two constructs (Q899S, D949S) did not express well enough to yield protein suitable for *in vitro* assays. For those proteins that were produced successfully, proper folding was verified by far-UV circular dichroism spectroscopy (Supporting Fig. S7C). When comparing binding of the di- and penta-arabinoside acceptor analogues (Fig. 4B and Fig. 5) that both carry the *O*-linked octyl tail, it was striking that the substitutions W868A and W985A affected binding of these ligands in a differential fashion. While the W985A mutation virtually abrogated binding of the disaccharide Ara(1→5)Ara-O-C8, the W868A substitution preserved binding of this particular ligand, with only a modestly higher *K_d_* (Table 2, Fig. 5A). In contrast, binding of the penta-arabinoside Ara(1→5)_4_Ara-O-C8 was insensitive to the W985A mutation, but completely inhibited in response to the W868A mutation. Likewise, mutating Asn740 to Ala weakened binding of the disaccharide (Table 2), consistent with its position within H-bond distance of the ordered Ara*f* in subdomain I, whereas the distant H911A mutation in subdomain II had no effect on this ligand. Thus, the differential effect of mutations in the putative binding sites in subdomain I and II on binding of acceptor analogues that differ only in length, strongly suggests that these bind preferentially to distinct sites on EmbC^CT^.

## Discussion

Polyprenyl-dependent glycosyltransferases of superfamily GT-C are still awaiting the determination of a structure of an intact, full-length enzyme, but structures of individual hydrophilic domains have begun to emerge [24] (see also PDB entry 3BYW). As a first step towards the complete structural characterisation of the Emb Ara*f*-transferases in *M. tuberculosis*, we have determined the crystal structure of the hydrophilic C-terminal domain of EmbC, the enzyme responsible for arabinan chain elongation in LAM synthesis and a target for the front line antibiotic EMB [5]. We found that the architecture of this domain comprises two subdomains, one of which folds as a lectin- or CBM-like domain, the other one shows weak similarity to the C-terminal hydrophilic domain of an unrelated GT-C glycosyltransferase, oligosaccharyl transferase STT3 [24]. The match between subdomain I and the so-called CC region of STT3 is poor (Fig. S4), and is limited to core secondary structure elements. Nevertheless, the DALI-derived superposition aligns the second Trp in STT3's highly conserved WWDYG motif with EmbC's Trp985, a side chain we showed is critical for enzymatic activity. Thus the alignment lends additional support to the notion of Trp985 sitting at a critical junction of the C-terminal domain of EmbC.

Sequence comparison of the Emb C-terminal domains (Fig. S1) strongly suggests that the disulfide bond Cys749-Cys993 is a conserved structural feature. Forming a topologically intuitive demarcation of this domain, this covalent link presumably enhances the stability of the C-terminal domain at physiological conditions in the host. The disordered loops (residues 794–825, 1016–1037) encompass regions of high sequence diversity as opposed to otherwise remarkably conserved regions of the structure. Given the latter, one could speculate that these disordered regions are linked to acceptor discrimination, and/or that ordering might be induced by contacts with adjacent structural elements in the context of the full-length enzyme.

It has previously been proposed that the Emb enzymes may function as dimers, possibly in the combination EmbA/EmbB and EmbC/EmbC [11], [28]. Our sedimentation velocity data now provide supporting evidence for self-assembly of EmbC, although we cannot rule out that the observed oligomerisation occurs solely as a result of separating EmbC^CT^ from the rest of the protein. However, the presence of dimers and trimers (or tetramers) (Fig. 4A) in solution demonstrated that at least two of the observed crystal packing interfaces were able to mediate self-assembly of EmbC^CT^. While thile the most-extended packing interface (SAS buried 1100 Å^2^) is mediated by structural elements (helices H0 and H6) that are close the truncation site, the second-largest interface (SAS buried 670 Å^2^) is mediated by strand S2, and distant to the truncation site. Indeed, the latter self-assembly interface generates a continuous β-sheet that extends across the monomer-monomer boundary (Fig. S5C), hinting that it could be preserved in the full-length enzyme.

The presence of a CBM-like subdomain in EmbC^CT^ is consistent the proposed role of the C-terminal domain in acceptor substrate recognition [10], [11]. Among these structurally diverse carbohydrate binding modules, the β-sandwich fold seen in EmbC^CT^ is most common [21]. The differential response of the ligands of different length to the Trp mutations in subdomains I and II provides compelling evidence for the presence of two separate ligand binding sites in EmbC^CT^. This response also links the loss of Ara*f* transferase activity in the Trp mutants to compromised acceptor binding. Although we were not successful in crystallising a complex structure that directly demonstrates binding of an acceptor analogue to the CBM-like subdomain II, the dramatic loss of binding affinity of the penta-arabinoside acceptor for the mutant EmbC^CT(W868A)^ (Fig. 5B, Table 2) and the corresponding loss of LAM synthesis, are strong indications that subdomain II indeed functions as a carbohydrate binding module. We note that the W868A mutation has also a modest effect on binding of Ara(1→5)Ara-O-C8 (∼2.5-fold increase in *K_d_*, Table 2), despite the obvious preference of this ligand for binding to subdomain I, as shown by the structure and the response to the W985A mutation. This observation could indicate that Ara(1→5)Ara-O-C8 also associate with the CBM-like subdomain II, albeit with considerably lower affinity. The converse may be true for the penta-saccharide as well, although the affinities we measured show no corresponding signature. Comparison of the affinities for binding of the tri- and pentasaccharide to wild type EmbC^CT^ clearly indicates that binding to subdomain II is tighter for longer polysaccharides, as these can be expected to make additional contacts. However, the apparent switch in binding preference from the site in subdomain I to that in subdomain II on going from two to five Ara*f* units is less straightforward to explain. If, as the structure suggests, only the octyl tail and the first Ara*f* unit were the major determinants of binding to subdomain I, one would expect to see evidence for binding of Ara(1→5)_4_Ara-O-C8 to subdomain I, that is, a significant change in affinity when mutating Trp985. Thus, while the octyl tail clearly influences binding of the di-saccharide, this appears to be less the case for the tri- and penta-saccharides. This observation is in line with the dispensable nature of the octyl chain when the above ligands are used as acceptor mimics in cell-free Ara*f* transferase assays [25].

Overall, a string of genetic and biochemical evidence consistently indicated that enzymatic activity of the Emb Ara*f*-transferases is associated with loops displayed on the extra-cellular face of the membrane. For instance, the most frequent point mutation present in EMB-resistant clinical isolates of *M. tuberculosis* concerns residue Met306 in EmbB ( = Met300 in EmbC, see Fig. 1) [20], only a few residues downstream of the GT-C-specific, strictly conserved DDX motif in the E2 loop [15]. Berg *et al.* showed that loop E6 carries a functionally relevant, conserved proline-containing sequence motif [10], consistent with findings in the Emb protein of *C. glutamicum* [14]. Moreover, a crystal structure of the first extracellular loop of the Emb Ara*f*-transferase of the related organism *Corynebacterium diphtheriae* has become available very recently (PDB entry 3BYW; Tan K., Hatzos C., Abdullah J., Joachimiak A., unpublished). The domain of the E1 loop displays a β-sandwich fold with similarity to the fold of galectin [29], but is not superimposable on that of subdomain II of EmbC^CT^. The galectin-like fold again hints to a potential function in carbohydrate binding – perhaps the sugar moiety of the Ara*f*-donor DPA. In conclusion, the present structure of the C-terminal domain of *M. tuberculosis* EmbC provides a first corner stone towards assembling the structure of the full-length enzyme, and allows us to begin probing this essential enzyme in a rational and targeted fashion.

## Methods

### Reagents

Plasmids were propagated during cloning in *E. coli* Top10 cells (Invitrogen). All restriction enzymes, T4 DNA ligase and Phusion DNA polymerase enzymes were sourced from New England Biolabs. Oligonucleotides were from MWG Biotech Ltd and PCR fragments were purified using the QIAquick gel extraction kit (Qiagen). Plasmid DNA was purified using the QIAprep purification kit (Qiagen).

### Recombinant protein

A 1125-bp region coding for the C-terminal domain (residues 719–1094) of EmbC was cloned from genomic DNA of *M. tuberculosis* H37Rv using PCR primers (restriction sites underlined) GATCGATCCATATGGAGGTGGTATCGCTGACCCAG (forward) and GATCGATCCTCGAGCTAGCCTCTGCGCAACGGC (reverse). The PCR product was ligated into plasmid pET23b (*Nde*I, *Xho*I restriction sites), yielding the His6-tagged pET23b-EmbC^CT^ construct, whose sequence was verified (School of Biosciences Genomics Facility, University of Birmingham). For expression, *E. coli* C41(DE3) cells were transformed with pET23b-EmbC^CT^ using the rubidium chloride method. Overnight cultures (5 ml LB medium, 100 µg/ml ampicillin) were used to inoculate bulk cultures (4×1 litre LB, 100 µg/ml ampicillin, 37°C, 200 rpm). Seleno-methionine derivatised EmbC^CT^ was produced using the same expression plasmid and host, but following the feedback inhibition protocol described in [30]. Cultures were induced at OD_600_ = 0.5 using 1 mM IPTG (12 h, 16°C). Cells were harvested (6000×*g*, 15 min), washed with 20 ml phosphate buffered saline, and frozen. Pellets were re-suspended in 50 mM KH_2_PO_4_ (pH 7.9), 300 mM NaCl, 1 mM PMSF, 15 µg/ml benzamidine, DNAse and RNAse (50 µg/ml), and sonicated (30 sec ON/OFF cycles, total of 8 cycles). The lysate was cleared (30 min, 28000×*g*, 4°C) and passed over a HiTRAP Ni^2+^-NTA column (GE Healthcare), equilibrated in 50 mM KH_2_PO_4_ (pH 7.9), 300 mM NaCl, and eluted using a step-gradient of 50–500 mM imidazole. The purification was monitored by 12% SDS-PAGE. Fractions containing EmbC^CT^ (250, 500 mM imidazole) were pooled and dialysed against 50 mM KH_2_PO_4_ (pH 7.9), 300 mM NaCl, and concentrated by ultrafiltration to ∼15 mg/ml.

### Structure determination

Hanging drop vapour diffusion was used to grow crystals of EmbC^CT^ over a reservoir of 0.1 M sodium acetate pH 4.4, 80 mM ammonium phosphate, mixing 1 µl of protein with 1 µl of reservoir solution. Crystals were cryoprotected in reservoir solution, adding up to 12% ethylene glycol and 12% glycerol, and flash frozen in liquid nitrogen. Native and 3-wavelength SeMet MAD data were recorded on beamline ID23-1 (ESRF, Grenoble, France). Diffraction images were processed using XDS and XSCALE [31] (Table 1). Selenium sites and phases were obtained using standard procedures (SHELXD [32], SHARP v2.2 [33] SOLOMON [34]) leading to a readily interpretable electron density map (Fig. S2A). The ARP/wARP-built [35] initial model was rebuilt in COOT [36], with intermittent refinement against native data (REFMAC5 [37], PHENIX.REFINE [38]). Temperature factor modelling included TLS refinement [39]. The final model has good stereochemistry and comprises EmbC residues 735–794, 825–1015 and 1038–1067, 113 water molecules, one molecule of Ara(1→5)Ara-O-C8, one Ca^2+^ and one phosphate ion (Table 1).

### Solution binding assay by intrinsic tryptophan fluorescence

Intrinsic tryptophan fluorescence (ITF) experiments were carried out using a PTI QuantaMaster 40 spectrofluorimeter, recording data with the FeliX32 software package (PTI, Birmingham, New Jersey, USA). The excitation wavelength was set to 294 nm and the fluorescence emission (*F_emission_*) was recorded between 300–400 nm for each ligand aliquot added to a 200 µl solution containing 20 µM EmbC^CT^ in 50 mM KH_2_PO_4_ (pH 7.9), 300 mM NaCl. For EmbC^CT^, the emission maximum (*F_emission_^max^*) was at λ = 338 nm, providing a basal *F_emission_* coordinate for the collection of subsequent ITF data. The change in fluorescence emission (*ΔF_emission_*) was calculated by subtracting *F_emission_* (recorded 2 min after each ligand addition) from *F_emission_^max^*, and the data was then plotted against ligand concentration, [L] (3 independent experiments). A plot of *ΔF_emission_* at λ = 338 nm vs. [L] was fitted to the saturation binding equation using GraphPad Prism software:
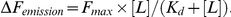



### Circular dichroism spectroscopy

Far-UV circular dichroism (CD) spectra were recorded at 25°C using a Jasco J-715 spectropolarimeter and a cell of 0.01 cm path length. Proteins EmbC^CT^, EmbC^CT(N740A)^, EmbC^CT(W868A)^, EmbC^CT(H911A)^ and EmbC^CT(W985A)^ were dialysed into 50 mM KH_2_PO_4_ (pH 7.9), 50 mM NaF to a final concentration of 0.5 mg/ml each. Spectra were recorded of 250 µl aliquots of each protein by measuring ellipticity from 195–260 nm, using a bandwidth of 2 nm and a scan speed of 100 nm/min. Spectra were normalised by subtracting the spectrum of buffer alone (baseline).

### Analytical ultracentrifugation

Sedimentation velocity experiments were performed using a Beckman Proteome XL-I analytical ultracentrifuge equipped with absorbance optics. EmbC^CT^ was dialysed into 50 mM KH_2_PO_4_ (pH 7.9), 300 mM NaCl, and loaded into cells with two channel Epon centre pieces and quartz windows. A total of 100 absorbance scans (280 nm) were recorded (40,000 rpm, 4°C) for each sample, representing the full extent of sedimentation of the sample. Data analysis was performed using the SEDFIT software, fitting a single friction coefficient [40].

### Generation of *embC*-deficient *M. smegmatis* and complementation plasmids

Approximately 1 kb of upstream and downstream flanking sequences of the *embC* gene (*MSMEG2785*) were PCR amplified from *M. smegmatis* mc^2^155 genomic DNA using the primer pairs MSEMBCLL, MSEMBCLR, MSEMBCRL and MSEMBCRR, respectively (sequences listed in Supporting Information Table S1). Following restriction digestion of the primer incorporated *AlwNI* sites, the PCR fragments were cloned into *AlwNI*-digested p0004S to yield the knockout plasmid pΔ*MSMEGEMBC* which was then packaged into the temperature sensitive mycobacteriophage phAE159 as described previously [27] to yield phasmid DNA of the knockout phage phΔ*MSMEGEMBC*. Generation of high titre phage particles and specialized transduction were performed as described earlier [27], [41]. Deletion of *MSMEGEMBC* in one hygromycin-resistant transductant was confirmed by Southern blot. For complementation, *M. tuberculosis embC* was cloned using primer pairs Mt-*embC*-forward and Mt-*embC*-reverse (sequences listed in Supporting Information Table S1) and blunt-end ligated into *Sma*I digested pUC18. For QuikChange mutagenesis (Stratagene) of pUC18-Mt-*embC* W868A and W985A codons, primer pairs W868A-sense/-antisense and W985A-sense/-antisense (sequences in Supporting Information Table S1, each with 5′-phosphate modifications) were used. The 3301 bp product was extracted from plasmids (pUC18-Mt-*embC*, pUC18-Mt-*embC* ^W868A^ and pUC18-Mt-*embC* ^W985A^) digested with *Nde*I and *Hind*III, and sub-cloned into the similarly digested mycobacterial shuttle vector pVV16 to yield pVV16-Mt-*embC*, pVV16-Mt-*embC* ^W868A^ and pVV16-Mt-*embC* ^W985A^. These plasmids were then used to transform *M. smegmatis*Δ*embC* to yield clones resistant to both hygromycin and kanamycin.

### Point mutations in recombinant EmbC^CT^

QuikChange mutagenesis (Stratagene) was carried out using pET23b-Mt-*embC^CT^* (generated as described above). Primer pairs used for the codon alterations N740A, W868A, Q899S, H911A and W985A are listed in the Supporting Information Table S1. Mutant plasmids were subsequently transformed individually into *E. coli* C41 (DE3). Mutant proteins were expressed and purified as described above.

### Analysis of lipoglycans

Lipoglycans form *M. smegmatis* strains were extracted as described previously [42]. Dried cells were resuspended in de-ionized water and disrupted by sonication (MSE Soniprep 150, 12 µm amplitude, 60 s on, 90 s off for 10 cycles, at 4°C). An equal volume of ethanol was added to the cell suspension and the mixture was refluxed at 68°C, for 12 h intervals, followed by centrifugation and recovery of the supernatant. The C_2_H_5_OH/H_2_O extraction process was repeated five times and the combined supernatants dried. The dried supernatant was then subjected to hot-phenol treatment by addition of phenol/H_2_O (80%, w/w) at 70°C for 1 h, followed by centrifugation and the aqueous phase was dialyzed using a 1500 MWCO membrane (Spectrapore) against de-ionized water. The retentate was dried, resuspended in water and sequentially digested with α-amylase, DNase, RNase, chymotrypsin and trypsin. The retentate was further dialyzed using a 1500 MWCO membrane (Spectrapore) against deionized water. The eluates were collected, extensively dialysed against deionized water, concentrated and analyzed by 15% SDS-PAGE using a Pro-Q emerald glycoprotein stain (Invitrogen).

### Accession numbers

The accession number for the coordinates and structure factors of the C-terminal domain of EmbC in the Protein Data Bank (http://www.rcsb.org) is 3PTY.

## Supporting Information

Figure S1 Sequence alignment of EmbC^CT^. CLUSTALW2-aligned sequences of the C-terminal domain of EmbC (residues 719–1094) and related Emb enzymes. Species names are abbreviated as Mt = *M. tuberculosis*, Ms = *M. smegmatis*, Cg = *C. glutamicum*. The sequence alignment was formatted using ESPript (espript.ibcp.fr, reference [43]). Dashed underlines indicate disordered region, orange and blue bars indicate subdomains I and II, respectively, and residue numbers refer to the sequence of *M. tuberculosis* EmbC.
(1.94 MB TIF)

Figure S2 Experimental electron density and Ca^2+^ site. A) Solvent-flattened electron density map, contoured at 1.2 σ, calculated based on the seleno-methionine substructure, and superimposed over the final refined model of EmbC^CT^ (yellow sticks). The region shown is the S10–S11 loop with the Ca^2+^ binding site. B) Comparison of σ_A_-weighted *F_o_−F_c_* density (contour level 4.5σ) without EDTA (green), and with 10 mM EDTA (purple) in the cryo-buffer. Density was calculated with phases and calculated amplitudes of a protein-only coordinate set. The height for the Ca^2+^ peak is 21σ (no EDTA) and 7σ (10 mM EDTA), respectively, while the height of the nearby phosphate peak is ∼7.5σ in both maps.
(3.06 MB TIF)

Figure S3 Comparison of subdomain II of EmbC^CT^ with structural neighbours. EmbC^CT^ (blue strands, green helices) superimposed over structural neighbours (yellow ribbons) identified by DALI, reference [23]. A) Carbohydrate binding module (CBM) of *Paenibacillus polymyxa* endo-1,4-β-xylanase (CBM family 36) in complex with β-D-xylopyranose trisaccharide (yellow sticks, 1UX7, reference [44]). B) CBM family 6: *Cellvibrio mixtus* cellulase B bound to a β-D-glucose trisaccharide (red sticks, 1UYY, reference [26]) C) CBM family 6: *Bacillus halodurans* BH0236 bound to xylobiose (red sticks, 1W9T, reference [45]). Bound Ca^2+^ ions are shown as spheres in green and magenta for EmbC^CT^ and the superimposed CBM, respectively. The side chain of Trp868 in the ‘outer’ β-sheet of EmbC^CT^ is shown in grey sticks.
(1.97 MB TIF)

Figure S4 Superposition of EmbC^CT^ with *Pyrococcus furiosus* STT3's C-terminal domain. Superposition of EmbC^CT^ with the ‘central core’ domain of the C-terminal hydrophilic domain of oligosaccharyltransferase *Pyrococcus furiosus* STT3 (yellow ribbon, reference [24]) calculated using DALI. Secondary structure elements of EmbC^CT^ with matches in STT3 are labelled in accordance to Figs. 2 and S1. Side chains of the catalytic WWDYG motif in STT3 and of the corresponding tryptophan residue in EmbC^CT^ (Trp985) are shown in blue and red sticks, respectively. The view in panel B is rotated by 90° about the vertical axis relative to panel A, and restricted to subdomain I (residues 735–759, 968–1067).
(1.19 MB TIF)

Figure S5 Major packing interfaces of the EmbC^CT^ crystal lattice. A) Arrangement of 3 copies of EmbC^CT^ on the crystal lattice around the two major packing interfaces, burying 1100 Å^2^ (green-magenta) and 670 Å^2^ (green-gray) of solvent-accessible surface (SAS) per monomer. B) The helix H0-mediated packing interface burying1100 Å^2^ SAS per monomer. C) The strand S2-mediated packing interface (670 Å^2^ SAS buried per monomer) demonstrating β-sheet formation across the interface.
(1.82 MB TIF)

Figure S6 Conformational changes in the ligand binding site between *apo* and Ara(1→5)Ara-O-C8-bound structures of EmbC^CT^. Blue and red density corresponds to contour levels of +3σ and −3σ, respectively, of a σ_A_-weighted *F_o_−F_c_* difference map calculated with phases and amplitudes *F_c_* of the *apo* model (cyan sticks) and observed amplitudes *F_o_* of the Ara(1→5)Ara-O-C8-bound structure (yellow sticks).
(1.06 MB TIF)

Figure S7 Mutations in EmbC, membrane incorporation of recombinant EmbC and CD analysis of EmbC^CT^ point mutants. A) Ribbon diagram of EmbC^CT^, with subdomains I and II shown with orange and blue β-strands, respectively. The Ara(1→5)Ara-O-C8 ligand (and one of its symmetry-related copies) are shown in grey sticks. The semi-transparent sticks show a β-D-Gal hexamer from the structural superposition of EmbC^CT^ with the family 6 CBM of β-agarase (PDB entry 2CDO, reference [46]). Mutated residues are indicated with their sequence numbers. B) Plasmids pVV16 encoding full-length EmbC, or point mutants thereof, were transformed into an *embC*-deficient *M. smegmatis*. Cell homogenates were separated into membrane (M) and cytosolic (C) fractions, and probed with an anti-His6 antibody (Roche). The lanes are as follows: 1 - pVV16 (empty vector), 2 - pVV16-Mt-*embC*, 3 - pVV16-Mt-*embC* ^W868A^, 4 - pVV16-Mt-*embC* ^W985A^. C) Far-UV circular dichroism spectra of recombinant EmbC^CT^ (wild-type and point mutants).
(1.28 MB TIF)

Table S1 Primer sequences used for mutagenesis.
(0.06 MB DOC)
