# Supplementary figures and images for: The C-Terminal Domain of the Arabinosyltransferase Mycobacterium tuberculosis EmbC Is a Lectin-Like Carbohydrate Binding Module
Source: PLoS Pathog. 2011 Feb 24;7(2):e1001299. doi: 10.1371/journal.ppat.1001299 (PMC3044687; doi:10.1371/journal.ppat.1001299)

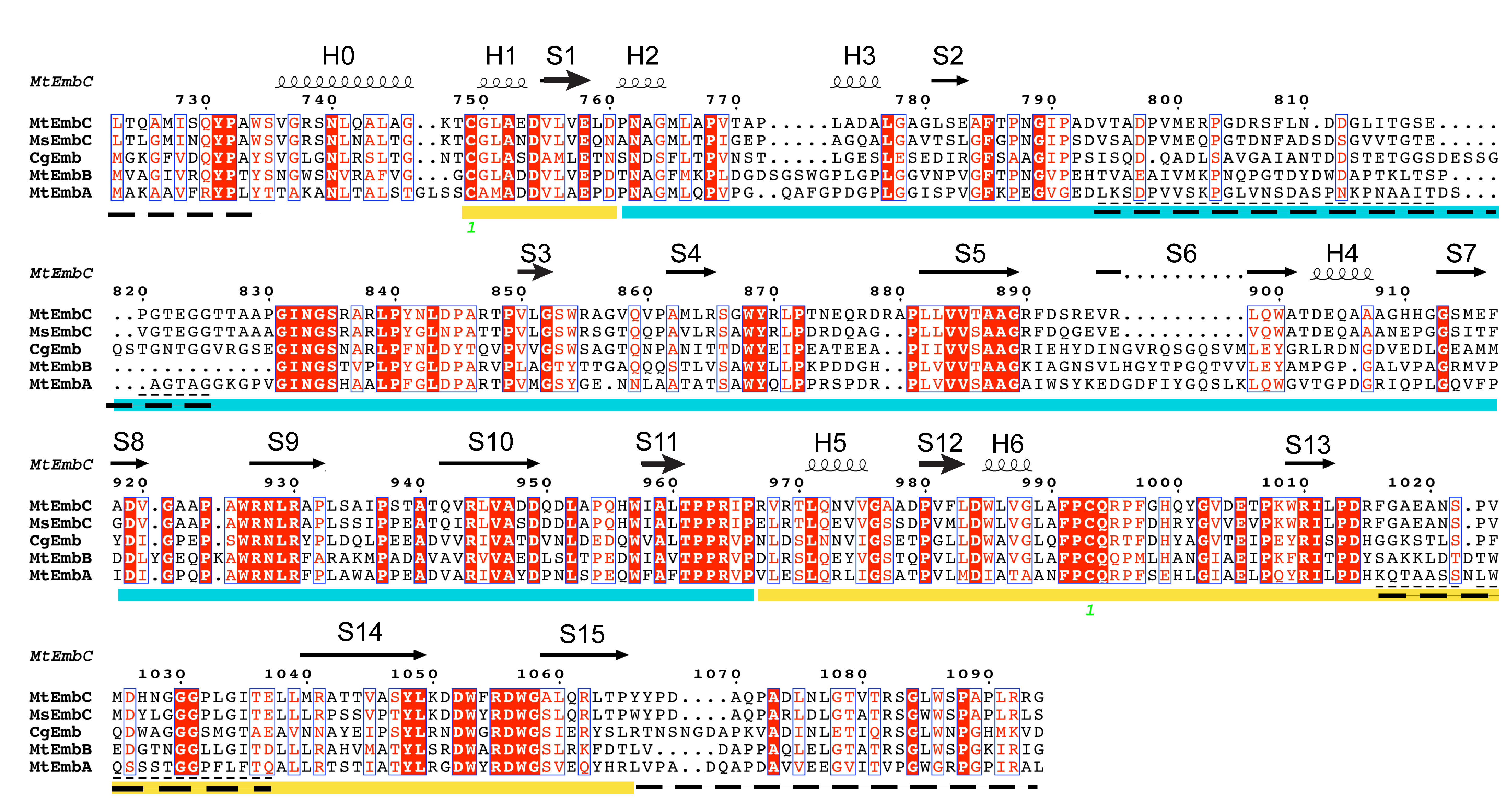

Supplement: Figure S1 — Sequence alignment of EmbCCT. CLUSTALW2-aligned sequences of the C-terminal domain of EmbC (residues 719–1094) and related Emb enzymes. Species names are abbreviated as Mt = M. tuberculosis, Ms = M. smegmatis, Cg = C. glutamicum. The sequence alignment was formatted using ESPript (espript.ibcp.fr, reference [43]). Dashed underlines indicate disordered region, orange and blue bars indicate subdomains I and II, respectively, and residue numbers refer to the sequence of M. tuberculosis EmbC. (1.94 MB TIF) [file ppat.1001299.s001.tif]

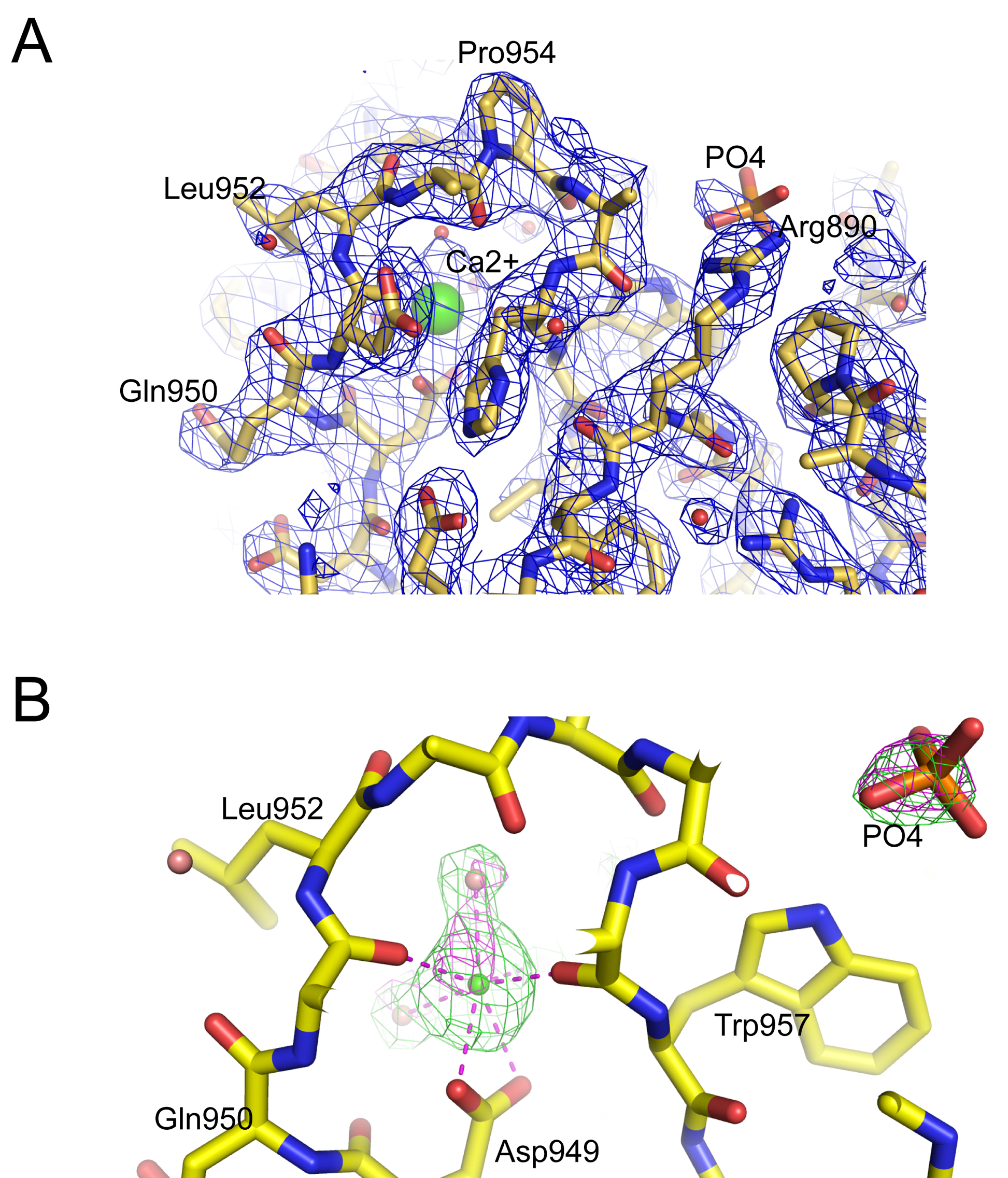

Supplement: Figure S2 — Experimental electron density and Ca2+ site. A) Solvent-flattened electron density map, contoured at 1.2 σ, calculated based on the seleno-methionine substructure, and superimposed over the final refined model of EmbCCT (yellow sticks). The region shown is the S10–S11 loop with the Ca2+ binding site. B) Comparison of σA-weighted Fo−Fc density (contour level 4.5σ) without EDTA (green), and with 10 mM EDTA (purple) in the cryo-buffer. Density was calculated with phases and calculated amplitudes of a protein-only coordinate set. The height for the Ca2+ peak is 21σ (no EDTA) and 7σ (10 mM EDTA), respectively, while the height of the nearby phosphate peak is ∼7.5σ in both maps. (3.06 MB TIF) [file ppat.1001299.s002.tif]

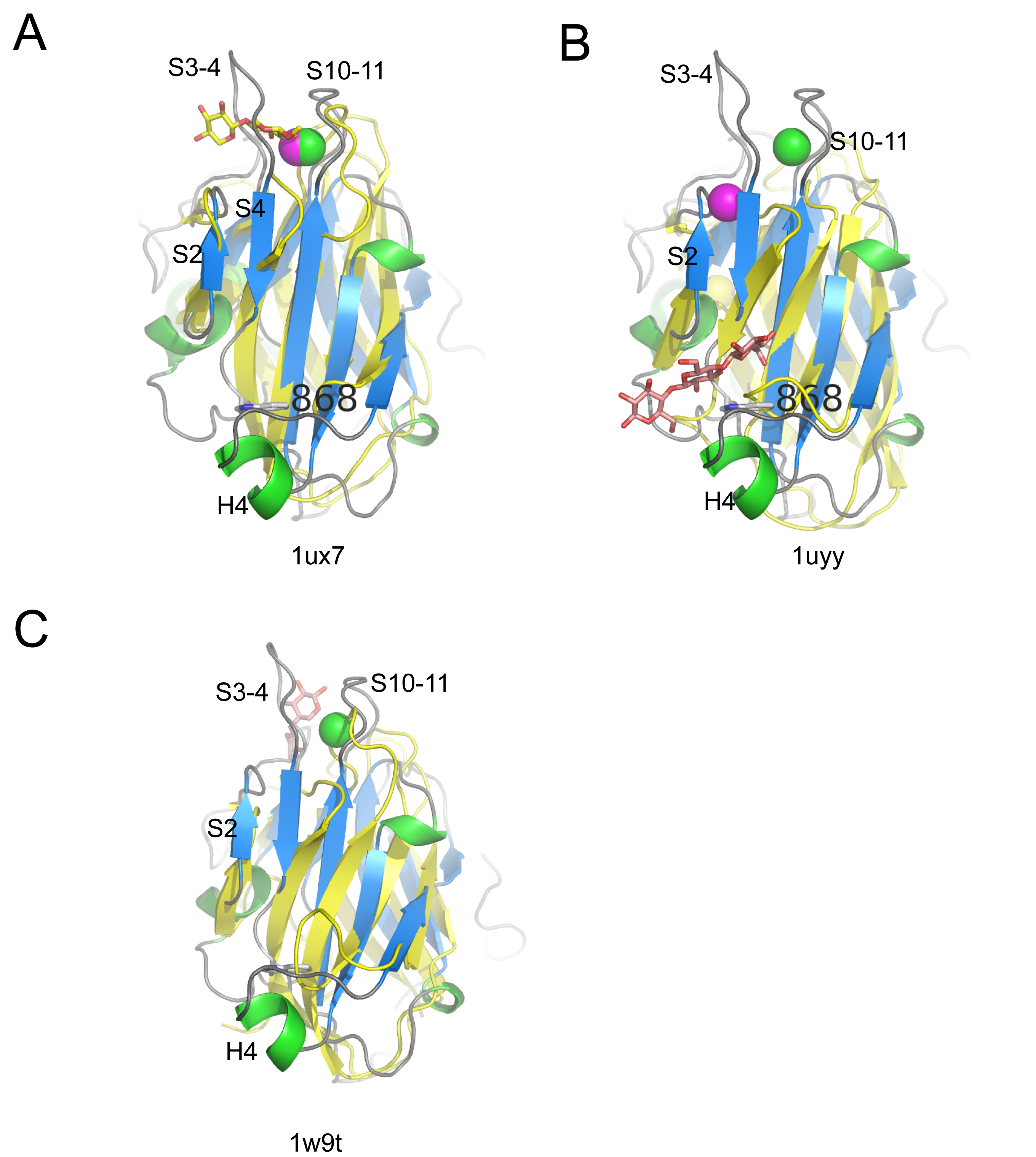

Supplement: Figure S3 — Comparison of subdomain II of EmbCCT with structural neighbours. EmbCCT (blue strands, green helices) superimposed over structural neighbours (yellow ribbons) identified by DALI, reference [23]. A) Carbohydrate binding module (CBM) of Paenibacillus polymyxa endo-1,4-β-xylanase (CBM family 36) in complex with β-D-xylopyranose trisaccharide (yellow sticks, 1UX7, reference [44]). B) CBM family 6: Cellvibrio mixtus cellulase B bound to a β-D-glucose trisaccharide (red sticks, 1UYY, reference [26]) C) CBM family 6: Bacillus halodurans BH0236 bound to xylobiose (red sticks, 1W9T, reference [45]). Bound Ca2+ ions are shown as spheres in green and magenta for EmbCCT and the superimposed CBM, respectively. The side chain of Trp868 in the ‘outer’ β-sheet of EmbCCT is shown in grey sticks. (1.97 MB TIF) [file ppat.1001299.s003.tif]

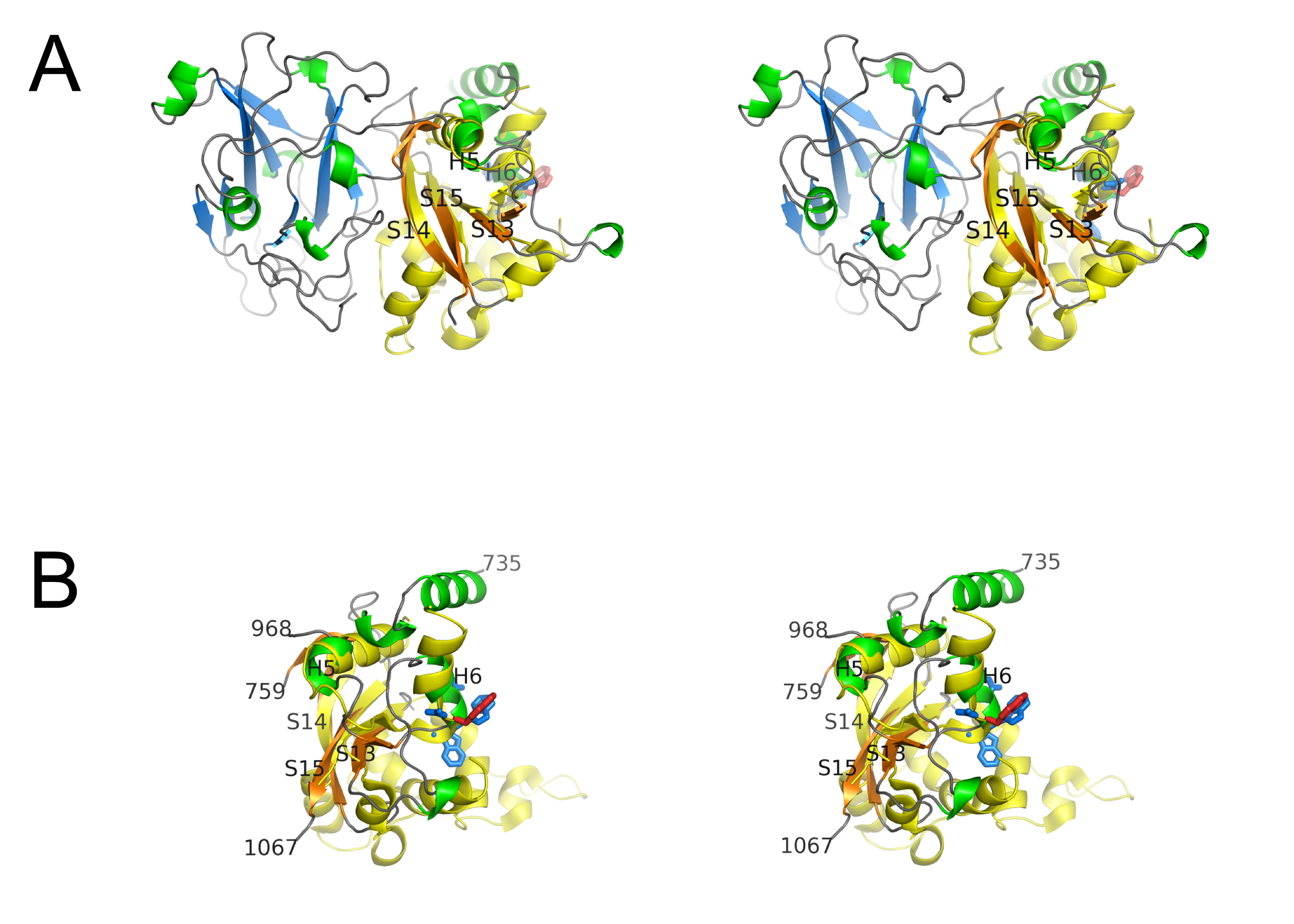

Supplement: Figure S4 — Superposition of EmbCCT with Pyrococcus furiosus STT3's C-terminal domain. Superposition of EmbCCT with the ‘central core’ domain of the C-terminal hydrophilic domain of oligosaccharyltransferase Pyrococcus furiosus STT3 (yellow ribbon, reference [24]) calculated using DALI. Secondary structure elements of EmbCCT with matches in STT3 are labelled in accordance to Figs. 2 and S1. Side chains of the catalytic WWDYG motif in STT3 and of the corresponding tryptophan residue in EmbCCT (Trp985) are shown in blue and red sticks, respectively. The view in panel B is rotated by 90° about the vertical axis relative to panel A, and restricted to subdomain I (residues 735–759, 968–1067). (1.19 MB TIF) [file ppat.1001299.s004.tif]

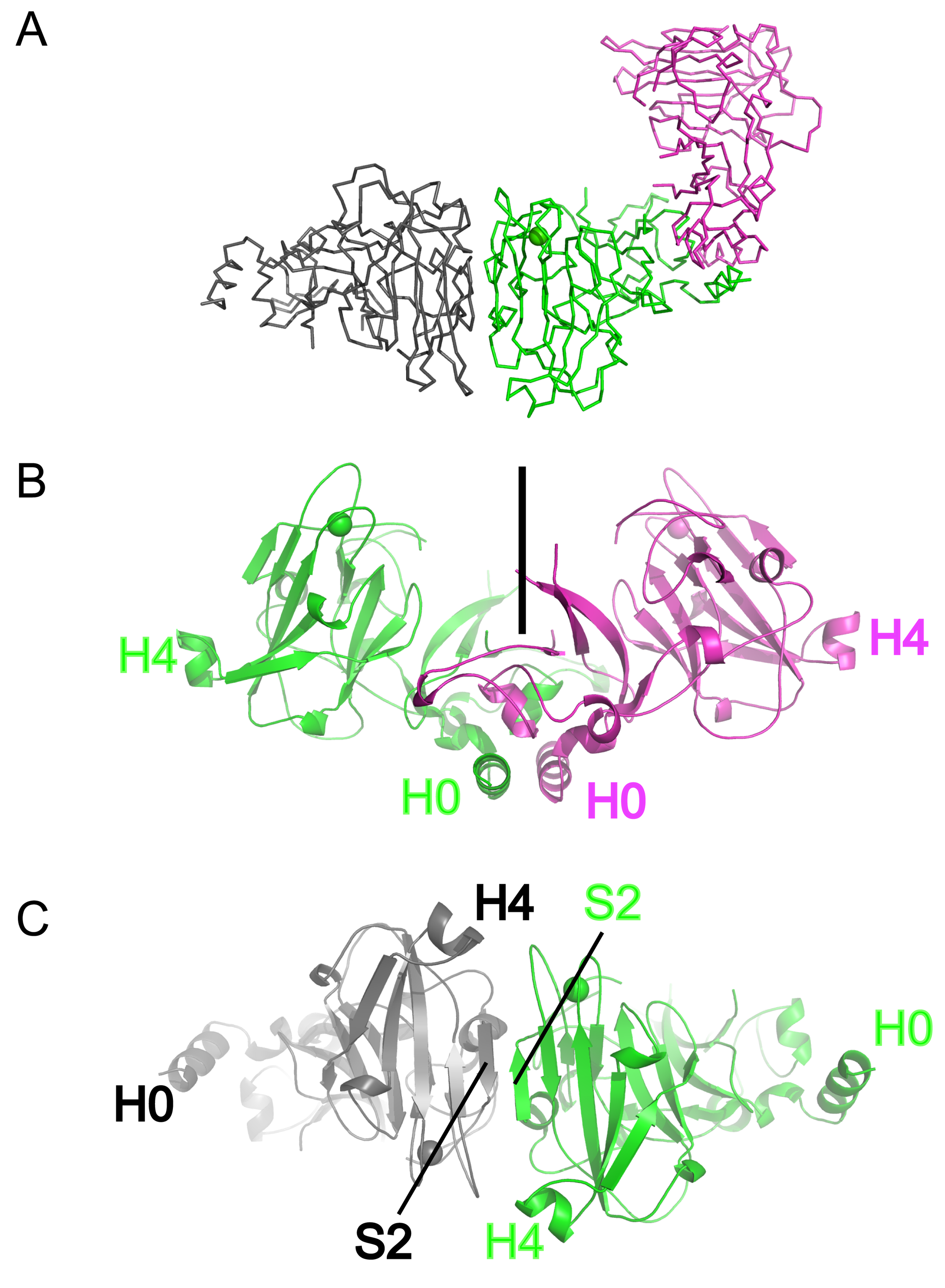

Supplement: Figure S5 — Major packing interfaces of the EmbCCT crystal lattice. A) Arrangement of 3 copies of EmbCCT on the crystal lattice around the two major packing interfaces, burying 1100 Å2 (green-magenta) and 670 Å2 (green-gray) of solvent-accessible surface (SAS) per monomer. B) The helix H0-mediated packing interface burying1100 Å2 SAS per monomer. C) The strand S2-mediated packing interface (670 Å2 SAS buried per monomer) demonstrating β-sheet formation across the interface. (1.82 MB TIF) [file ppat.1001299.s005.tif]

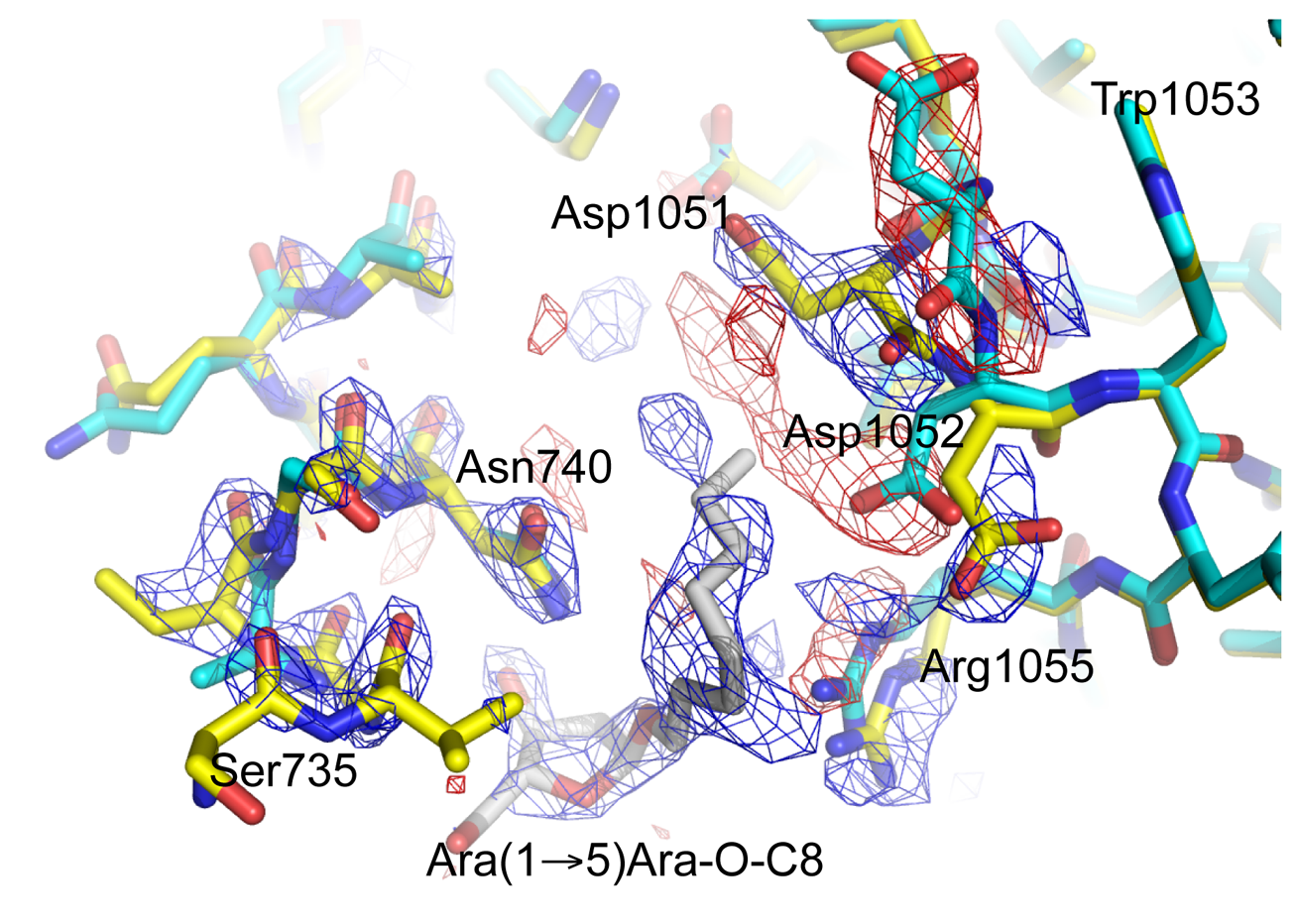

Supplement: Figure S6 — Conformational changes in the ligand binding site between apo and Ara(1→5)Ara-O-C8-bound structures of EmbCCT. Blue and red density corresponds to contour levels of +3σ and −3σ, respectively, of a σA-weighted Fo−Fc difference map calculated with phases and amplitudes Fc of the apo model (cyan sticks) and observed amplitudes Fo of the Ara(1→5)Ara-O-C8-bound structure (yellow sticks). (1.06 MB TIF) [file ppat.1001299.s006.tif]

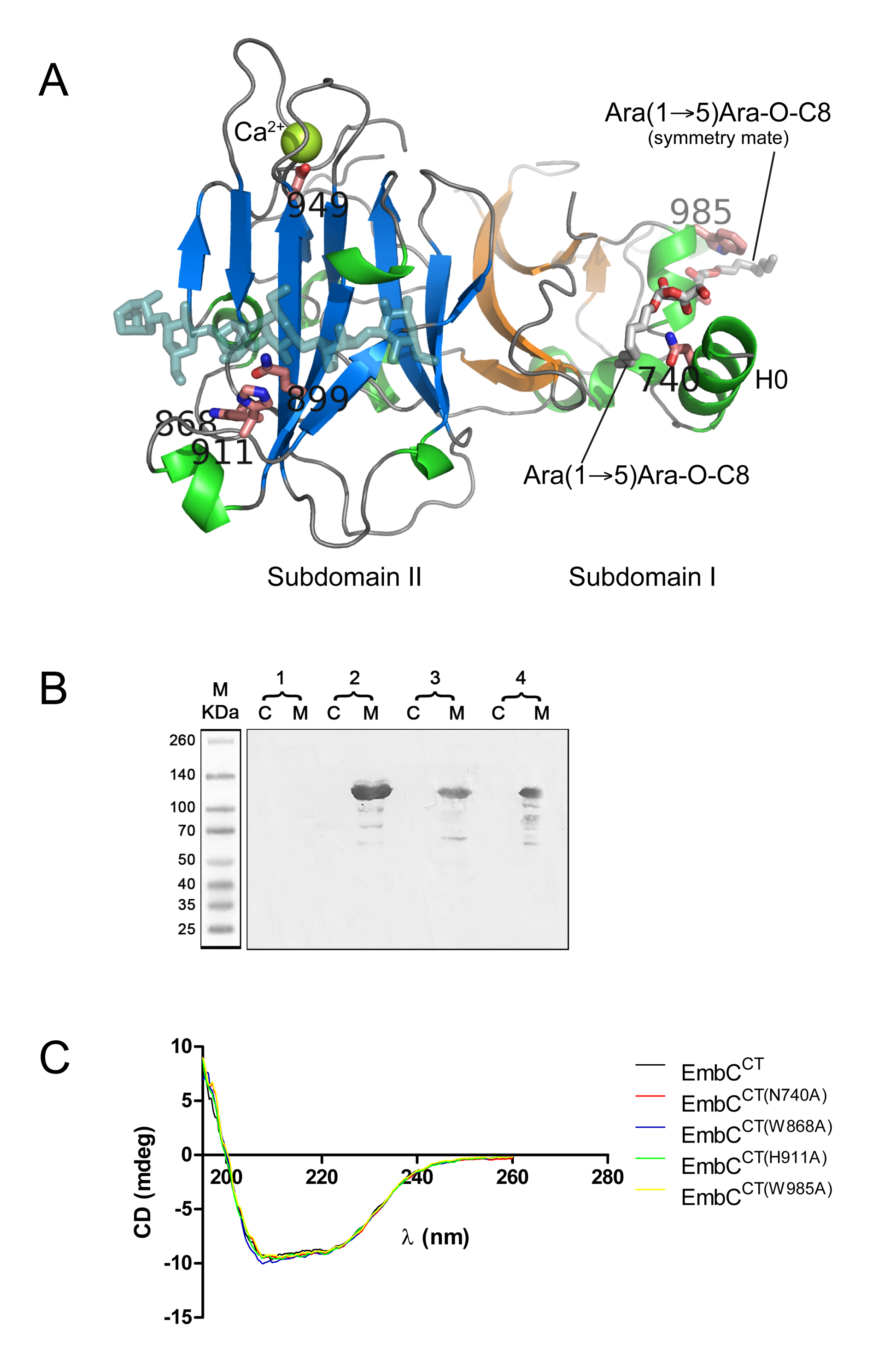

Supplement: Figure S7 — Mutations in EmbC, membrane incorporation of recombinant EmbC and CD analysis of EmbCCT point mutants. A) Ribbon diagram of EmbCCT, with subdomains I and II shown with orange and blue β-strands, respectively. The Ara(1→5)Ara-O-C8 ligand (and one of its symmetry-related copies) are shown in grey sticks. The semi-transparent sticks show a β-D-Gal hexamer from the structural superposition of EmbCCT with the family 6 CBM of β-agarase (PDB entry 2CDO, reference [46]). Mutated residues are indicated with their sequence numbers. B) Plasmids pVV16 encoding full-length EmbC, or point mutants thereof, were transformed into an embC-deficient M. smegmatis. Cell homogenates were separated into membrane (M) and cytosolic (C) fractions, and probed with an anti-His6 antibody (Roche). The lanes are as follows: 1 - pVV16 (empty vector), 2 - pVV16-Mt-embC, 3 - pVV16-Mt-embC W868A, 4 - pVV16-Mt-embC W985A. C) Far-UV circular dichroism spectra of recombinant EmbCCT (wild-type and point mutants). (1.28 MB TIF) [file ppat.1001299.s007.tif]
