# Supplementary material for: The C-Terminal Domain of the Arabinosyltransferase Mycobacterium tuberculosis EmbC Is a Lectin-Like Carbohydrate Binding Module
Source: PLoS Pathog. 2011 Feb 24;7(2):e1001299. doi: 10.1371/journal.ppat.1001299 (PMC3044687; doi:10.1371/journal.ppat.1001299)
Supplement: Table S1 — Primer sequences used for mutagenesis. (0.06 MB DOC) [file ppat.1001299.s008.doc]

# The C-terminal domain of the arabinosyltransferase *Mycobacterium tuberculosis* EmbC is a lectin-like carbohydrate binding module.

Luke J Alderwick^1^, Georgina S. Lloyd, Hemza Ghadbane^1^, John W. May^1^, Apoorva Bhatt^1^, Lothar Eggeling^2^, Klaus Fütterer^1,*^, Gurdyal S. Besra^1,*^

^1^School of Biosciences, University of Birmingham, Edgbaston, Birmingham B15 2TT, UK.

^2^Institut für Biotechnologie I, Forschungszentrum Jülich, D-52425 Jülich, Germany

*Contact: (KF) Email K.Futterer@bham.ac.uk; Phone +44 – 121 – 414 5895; Fax +44 – 121 – 414 5925

(GSB) Email G.Besra@bham.ac.uk; Phone +44 – 121 – 415 8125; Fax +44 – 121 – 414 5925

# Supporting Information

## Table S1. Primer sequences used for mutagenesis

| **Primers to generate knock-out plasmid p*ΔMSMEGEMBC*** |  |
| --- | --- |
| MSEMBCLL | 5'-TTTTTTTTCAGAAACTGGTGGCCGTGGTCGCGGTGTACGCG |
| MSEMBCLR | 5'-TTTTTTTTCAGTTCCTGGTTGGGATACACGTCGGATGCCAG |
| MSEMBCRL | 5'-TTTTTTTTCAGAGACTGCACGCCTGATCGCTGTCGTCGCCG |
| MSEMBCRR | 5'-TTTTTTTTCAGCTTCTGCACGCCTGATCGCTGTCGTCGCCG |
|  | Underlined sequence = *AlwN*I restriction sites |
|  |  |
| **Primers to generate complementation plasmid pVV16-Mt-*embC*** |  |
| Mt-*embC* -forward | 5'-GATCGATCCATATGGCTACCGAAGCCGCCCCACCCCGT |
| Mt-*embC* -reverse | 5'-GATCGATCAAGCTTGCCGCGGCGCAACGGCGCCGGACT |
|  | Underlined sequence = *Nde*I (forward) *HindIII* (reverse) restriction sites |
|  |  |
| **'QuikChange' primers to mutate pUC18-Mt-*embC and* pET23b-Mt-*embC^CT^*** |  |
| W868A-sense | 5'-GCCATGCTGCGGTCGGGCGCGTACCGGCTGCCCACCAAC |
| W868A-antisense | 5'-CGGTACGACGCCAGCCCGCGCATGGCCGACGGGTGGTTG |
| W985A-sense | 5'-GATCCGGTGTTCCTGGACGCGCTGGTGGGGCTGGCATTC |
| W985A-antisense | 5'-CTAGGCCACAAGGACCTGCGCGACCACCCCGACCGTAAG |

| **'QuikChange' primers to mutate pET23b-Mt-*embC^CT^*** |  |
| --- | --- |
| N740A-sense | 5’-TGGTCGGTTGGCCGGTCTGCCCTACAGGCTTTGGCCGGC |
| N740A-antisense | 5’-ACCAGCCAACCGGCCAGATTGGATGTCCGAAACCGGCCG |
| Q899S-sense | 5’-TCCCGCGAGGTCCGGTTGCAGTGGGCCACCGACGAGCAA |
| Q899S-antisense | 5’-AGGGCGCTCCAGGCCAACGTCACCCGGTGGCTGCTCGTT |
| H911A-sense | 5’-CAAGCGGCCGCCGGACACCACGGTGGGTCGATGGAATTC |
| H911A-antisense | 5’-GTTCGCCGGCGGCCTGTGGTGCCACCCAGCTACCTTAAG |
| D949S-sense | 5’-GTCCGGTTGGTCGCCGACGACCAGGATCTGGCGCCGCAG |
| D949S-antisense | 5’-CAGGCCAACCAGCGGCTGCTGGTCCTAGACCGCGGCGTC |
